# Chronotype and subthreshold bipolar disorder: emotional dysregulation and affective temperaments in the Italian general population

**DOI:** 10.1192/j.eurpsy.2026.10704

**Published:** 2026-07-31

**Authors:** G. Pontoni, S. Caiolo, A. Miola, A. Sarzetto, F. Sambataro

**Affiliations:** 1 Psychophysiological Selection Office, Psychiatry Section, Italian Army National Recruitment and Selection Center, Foligno (PG); 2 Psychiatry Section, Military Department of Forensic Medicine; 3 University of Padova, Padova, Italy; 4 Department of Neuroscience (DNS), Padua Neuroscience Center, University of Padova, Padova; 5 Vita-Salute San Raffaele University, Milano, Italy

## Abstract

**Introduction:**

Growing evidence supports an association between chronotype and mental health. In particular, the evening chronotype (ET) has been associated with increased vulnerability to bipolar disorder (BD).

**Objectives:**

This study aimed to estimate the prevalence of the different chronotypes in the general population, confirm the association between ET and subthreshold BD, clarify whether chronotype is associated with BD-related psychopathological dimensions and identify factors associated with subthreshold BD in the general population.

**Methods:**

A self-administered questionnaire including the reduced Morningness Eveningness Questionnaire (rMEQ), Brief-TEMPS-M, Difficulties in Emotion Regulation Scale (DERS), MDQ Mood disorder Questionnaire (MDQ) and Hypomania Check List-32, second revision (HCL-32-R2) scales was distributed to individuals attending of different Covid-19 vaccination centers and Covid-19 points of care.

**Results:**

A total of 2031 participants from the general population were enrolled. Overall, 22.8% of subjects had an evening chronotype (ET), 22.2% had a morning type (MT), and 55.0% displayed neither chronotype (NT). Significantly higher scores for depressive, anxious, irritable and cyclothymic affective temperament, along with a significant emotional dysregulation, were found in subjects with ET chronotype compared with MT and NT groups. The rMEQ score was significantly correlated with both the Brief-Temps M and the DERS scores. ET chronotypes exhibited significantly higher scores on both the MDQ and HCL-32-R2 scales, and a significant correlation was found between such scales and the rMEQ. ET chronotype and tobacco consumption were the only two factors, among those in Image 1, predicting subthreshold BD on both MDQ (Geneva and Hirschfeld scores) and HCL-32-R2 scales through a multivariate logistic regression model. In the same model, a good or excellent sleep quality was found to be a significant protector from subthreshold BP on both MDQ and HCL-32-R2 scales.

**Image 1:**
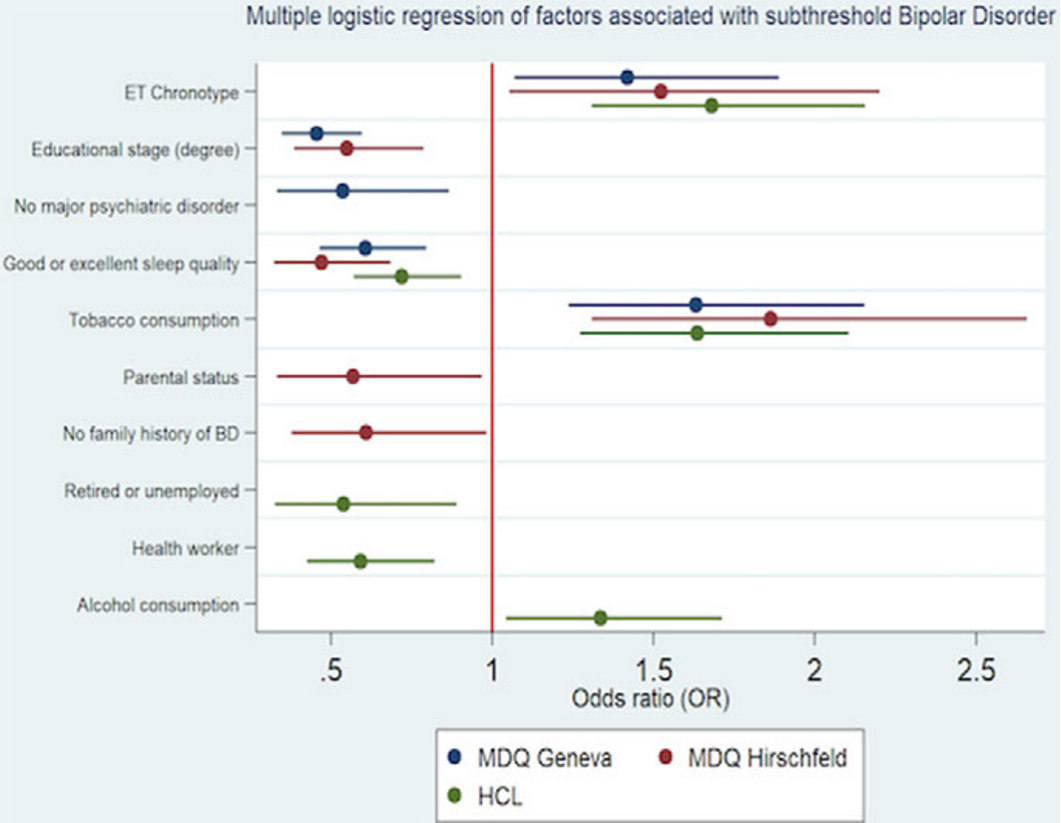
Image 1: Long description.

**Conclusions:**

Our findings strongly support an association between the ET chronotype and specific affective temperaments. Moreover, individuals with an evening chronotype are more likely to exhibit significant emotional dysregulation and an increased vulnerability to BD.

**Disclosure of Interest:**

None Declared

## Long descriptions

### Image 1: Long description

A forest plot titled Multiple logistic regression of factors associated with subthreshold Bipolar Disorder. The x-axis represents Odds ratio O R ranging from 0.5 to 2.5 with a vertical red reference line at 1.0. The y-axis lists ten factors. Data is categorized by three markers: blue dots for M D Q Geneva, red dots for M D Q Hirschfeld, and green dots for H C L.

* E T Chronotype: All three markers are above 1.0, ranging from approximately 1.4 to 1.7.

* Educational stage (degree): Blue and red markers are below 1.0, centered around 0.5.

* No major psychiatric disorder: Blue marker is below 1.0, centered around 0.6.

* Good or excellent sleep quality: Blue and red markers are below 1.0, while the green marker is slightly above 0.7.

* Tobacco consumption: All three markers are above 1.0, ranging from 1.6 to 1.9.

* Parental status: Red marker is below 1.0, centered at 0.6.

* No family history of B D: Red marker is below 1.0, centered at 0.6.

* Retired or unemployed: Green marker is below 1.0, centered at 0.5.

* Health worker: Green marker is below 1.0, centered at 0.6.

* Alcohol consumption: Green marker is above 1.0, centered at 1.3.

Horizontal lines through each dot represent confidence intervals.

Navigate back to Image 1:.

